# Polydactyly in the mouse mutant *Doublefoot* involves altered Gli3 processing and is caused by a large deletion in *cis* to *Indian hedgehog*

**DOI:** 10.1016/j.mod.2008.01.001

**Published:** 2008-05

**Authors:** Christian Babbs, Dominic Furniss, Gillian M. Morriss-Kay, Andrew O.M. Wilkie

**Affiliations:** a Weatherall Institute of Molecular Medicine, University of Oxford, John Radcliffe Hospital, Oxford, OX3 9DS, UK; b Department of Physiology, Anatomy and Genetics, South Parks Road, Oxford OX1 3QX, UK

**Keywords:** Shh, Ihh, Digit formation, Limb patterning

## Abstract

The mouse mutant *Doublefoot* (*Dbf*) shows preaxial polydactyly with 6–9 triphalangeal digits in all four limbs and additional abnormalities including a broadened skull, hydrocephalus, and a thickened, kinked tail. The autopod undergoes a characteristic expansion between late embryonic day (E) 10.5 and E11.5, following the onset of ectopic *Indian hedgehog* (*Ihh*) expression in the entire distal mesenchyme, except for the zone of polarising activity (ZPA), at E10.5. We show here that limb prepattern, as indicated by expression of *Gli3* and *Hand2* at E9.5 is unaffected by the mutation. As both *Sonic hedgehog* (*Shh*) and *Ihh* expression are present in *Dbf* limb buds at E10.5, we generated *Dbf*/^+^;*Shh^−/−^* mutants to analyse the effects of different patterns of Hedgehog activity on the limb phenotype and molecular differentiation. *Dbf*/^+^ embryos lacking *Shh* showed postaxial as well as preaxial polydactyly, and the *Ihh* expression domain extended posteriorly into the domain in which *Shh* is normally expressed, indicating loss of ZPA identity. Differences in gene expression patterns in wild type, single and compound mutants were associated with differences in Gli3 processing: an increased ratio of Gli3 activator to Gli3 repressor was observed in the anterior half of *Dbf*/^+^ limb buds and in both anterior and posterior halves of compound mutant limb buds at E10.5. To identify the cause of *Ihh* misregulation in *Dbf/^+^* mutants, we sequenced ∼20 kb of genomic DNA around *Ihh* but found no pathogenic changes. However, Southern blot analysis revealed a ∼600 kb deletion disrupting or deleting 25 transcripts, starting 50 kb 5′ of *Ihh* and extending away from the gene. The large deletion interval may explain the wide range of abnormalities in *Dbf*/^+^ mutants. However, we did not detect anologous deletions in cases of Laurin–Sandrow syndrome, a human disorder that shows phenotypic similarities to *Dbf.*

## 1 Introduction

The *Dbf* mutant, which arose spontaneously in the 3H1 (C3H/HeH × 101/H F1 hybrid) genetic background at Harwell (UK), is a polydactylous mouse that exhibits semidominant inheritance. Mice heterozygous or homozygous for *Dbf* have 6–9 digits in all four limbs; the extra digits are all triphalangeal and arise preaxially (Lyon et al., 1996; Hayes et al., 1998a). *Dbf*/^+^ mice also show malformation of the tibia, a broadened skull, hydrocephalus, a thickened kinked tail, and reduced fertility and viability. Homozygotes additionally exhibit a midline facial cleft but cannot be recovered alive beyond embryonic day (E) 14.5.

Polydactyly has been described in many mouse mutants, all except two of which show a discrete anterior domain of *Sonic hedgehog* (*Shh*) expression (Masuya et al., 1995; Hill et al., 2003). The Extra-toes (*Xt*^J^) mutant has an extended *Shh* domain due to functional inactivation of *Gli3* (Hui and Joyner, 1993), whereas *Dbf* mice exhibit ectopic *Indian hedgehog* (*Ihh*) expression in the distal limb bud mesenchyme (Yang et al., 1998). Ectopic *Ihh* upregulation is first detectable at E10.5 (Crick et al., 2003), the stage at which hyperexpansion of the autopod begins; downstream targets of *Shh* signalling are ectopically up-regulated (Hayes et al., 1998b; Yang et al., 1998). However, the molecular mechanism by which the polydactyly arises from ectopic *Ihh* expression has not been investigated.

The polydactylous phenotype of the *Xt*^J^ mutant was originally thought to result from the enlarged *Shh* expression domain (Hui and Joyner, 1993). However, *Shh^−^*^/^*^−^*;*Gli3^−^*^/^*^−^* mutants exhibit polydactyly in a similar pattern to *Gli3^−^*^/^*^−^* mutants, suggesting that the polydactyly of *Gli3*-deficient mice is independent of *Shh* (te Welscher et al., 2002). In wild type (wt) limb buds, digital number and identity are regulated by interaction between Shh and Gli3 (Litingtung et al., 2002). In the presence of Shh, Gli3 remains as a 190 kDa activator species, Gli3A, that up-regulates Hedgehog (Hh)-responsive gene expression, while in the absence of Shh, Gli3A is processed to a smaller 83–86 kDa repressor form, Gli3R, which negatively regulates expression of *Shh* and its target genes (Dai et al., 1999; Shin et al., 1999; Sasaki et al., 1999). Litingtung et al. (2002) suggested that in wt limb buds the Gli3A:Gli3R ratio controlled by *Shh* limits the polydactylous potential of the autopod, imposing pentadactyl constraint. This is supported by the localization of Shh protein in wt limb buds, which extends anterior to the zone of polarising activity (ZPA) in a domain coincident with *Patched1* (*Ptc1*) expression (Gritli-Linde et al., 2001), resulting in a posterior-to-anterior increase of the Gli3A:Gli3R ratio (Wang et al., 2000). Consistent with these observations, the Gli3 present throughout *Shh^−^*^/^*^−^* limb buds is mainly processed to Gli3R (Litingtung et al., 2002). Recently, the mutation underlying the polydactylous chicken *talpid*^3^ mutant has been reported to be in a novel gene and has also been shown to result in abnormal Gli3 processing (Davey et al., 2006). Given the evidence of involvement of abnormal Gli3 processing in the *Xt*^J^, *Shh^−^*^/^*^−^* and *talpid*^3^ mutants, it is possible that the polydactyly present in *Dbf* mice also results from aberrant Gli3 processing. This hypothesis is supported by evidence that Gli3 acts downstream of Ihh during endochondral skeletal development (Hilton et al., 2005; Koziel et al., 2005).

To investigate the mechanism by which polydactyly arises in *Dbf* we have analysed gene expression in *Dbf*/^+^ limbs, where there is an excess of Hedgehog (Hh) signalling, and compared this to *Shh^−^*^/^*^−^* limbs, where there is none. Since *Shh* and *Dbf* are located on different chromosomes (5 and 1, respectively) (Blake et al., 2003; Hayes et al., 2001), we have been able to generate mutant mice that carry two copies of the disrupted *Shh* allele and are heterozygous for the *Dbf* mutation. To further dissect the mechanisms underlying the limb malformations in both *Shh* and *Dbf* mutants, we have analysed the effects of the ectopic *Ihh* expression associated with *Dbf* limb abnormalities in the *Shh*-null background by correlating altered patterns of gene expression with the phenotype of single and double mutants. Differences in Gli3 processing between each genotype suggest that Hh-Gli3 interactions govern the observed differences in digital number, and that postaxial polydactyly results from expression of *Ihh*, but not *Shh*, in the posterior ZPA mesenchyme.

Previous attempts to identify the *Dbf* mutation have been unsuccessful. Hayes et al. (2001) constructed a high resolution genetic map and localized the mutation to a 0.4 cM interval on mouse chromosome 1. This region contained 35 genes including several plausible candidates for the *Dbf* mutation. However, despite the sequencing of three of these genes, the *Dbf* mutation remained unidentified. Based on the misregulation of *Ihh* expression in *Dbf*, we sequenced ∼20 kb of the surrounding genome but found no obvious pathogenic changes. To investigate whether a genomic rearrangement could be responsible, we used the mouse genome sequence to design a Southern blotting strategy to systematically screen the regions 5′ and 3′ of *Ihh* for copy number changes. We identified a ∼600 kb deletion starting ∼50 kb 5′ of *Ihh*, which removes or interrupts 25 known and predicted transcripts. This raises the possibility that additional abnormalities seen in *Dbf*/*Dbf* mice arise from loss of function of deleted genes, in addition to *Ihh* misregulation.

## 2 Results

### 2.1 The prepattern of *Dbf* limb buds is unaffected

**Fig. 1:**
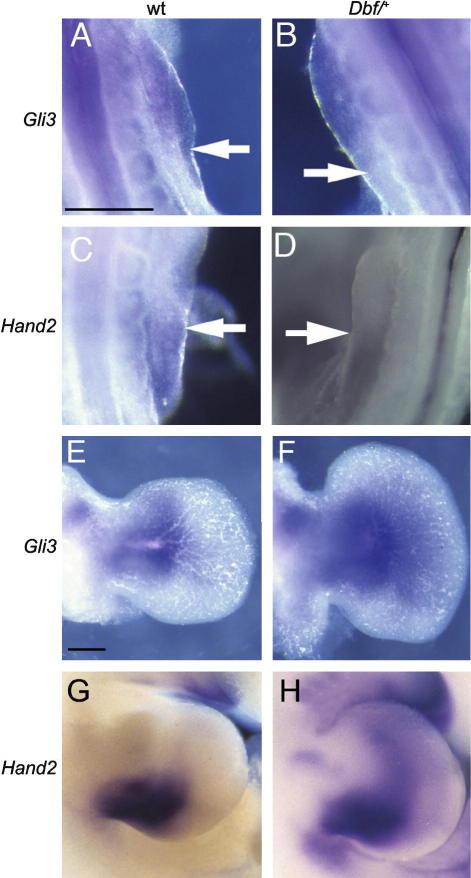
Expression of *Gli3* and *Hand2* in E9.5 (A–D) and E11.5 (E–H) **wt** (A, C, E, G) and *Dbf*/^+^ (B, D, F, H) forelimb buds. *Gli3* is expressed in the anterior region of both wt and *Dbf*/^+^ at E9.5, and *Hand2* in the posterior region (arrows indicate the limits of the expression domains). At E11.5 *Gli3* is expressed in a broader anterior domain in *Dbf*/^+^ limb buds, and *Hand2* expression extends more anteriorly in *Dbf*/^+^ than in equivalently staged **wt** limb buds. Scale bars 0.2 mm.

Expression of *Hand2* and *Gli3* has been implicated in patterning the limb bud prior to *Shh* expression, and has been shown to be affected later by the absence of *Shh* (Chiang et al., 2001; te Welscher et al., 2002). We assayed expression of these two genes before (E9.5) and after (E11.5) the onset of ectopic *Ihh* at E10.5 in *Dbf*/^+^ mutant embryos (Fig. 1). *Gli3* expression is restricted to the anterior portion of the limb bud in wt embryos at E9.5 (Fig. 1A) and this expression pattern is not altered in the limb buds of *Dbf*/^+^ mutants (Fig. 1B). *Hand2* is expressed throughout the flank of wt embryos prior to formation of the limb bud, then becomes limited to the posterior region of the limb bud as it is initiated (Fig. 1C); this pattern is not altered in *Dbf*/^+^ embryos at E9.5 (Fig. 1D). At E11.5, expression of *Gli3* in *Dbf*/^+^ limb buds differs from that in wt embryos in extending more distally; the domain is also broader although this probably simply reflects the greater breadth of the limb bud (Fig. 1F). Expression of *Hand2* is limited to the proximal posterior margin in wt E11.5 limb buds (Fig. 1G); in contrast, the *Hand2* domain in *Dbf*/^+^ limb buds extends anteriorly and distally (Fig. 1H). Hence the limb prepattern as indicated by the expression of *Hand2* and *Gli3* at E9.5 is unaffected in *Dbf*/^+^ limb buds, but the expression domains of both genes are altered in association with the presence of ectopic *Ihh* expression at E11.5 (Fig. 3H).

### 2.2 Altered limb phenotype of *Dbf* mutants in the absence of *Shh*

**Fig. 2:**
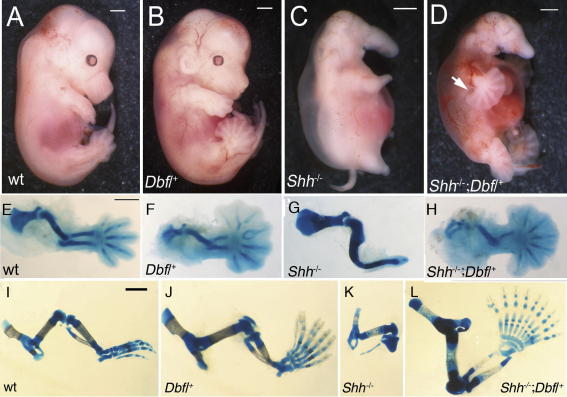
Morphology of E13.5 (A–H) and E17.5 (I–L) embryos and limbs, genotypes as indicated. (A and B) The preaxial polydactyly characteristic of *Dbf*/^+^ embryos (B) can be seen clearly in the hindlimb, which has 8 digits. (C) *Shh^−^*^/^*^−^* embryos show holoprosencephaly, oedema and highly truncated limbs. (D) *Shh^−^*^/^*^−^*;*Dbf*/^+^ embryos show only minor modification of the *Shh^−^*^/^*^−^* head phenotype; the limbs resemble those of *Dbf*/^+^ embryos except that the autopods show both preaxial and postaxial polydactyly; the whole embryo shows hypervascularization. (E–H) Cartilage preparations of E13.5 forelimbs. (E) wt forelimb, (F) *Dbf*/^+^ forelimb showing preaxial polydactyly. (G) The forelimb zeugopod and autopod of *Shh^−^*^/^*^−^* fetuses are represented by single elements and there is no elbow joint. (H) In the *Shh^−^*^/^*^−^*;*Dbf*/^+^ forelimb an elbow joint and radius are visible. (I) Wild type E17.5 hindlimb. (J) *Dbf*/^+^ hindlimb showing preaxial polydactyly (7 triphalangeal digits) and medial rotation (luxation) of the autopod due to the hypoplastic tibia. (K) Hindlimb of *Shh^−^*^/^*^−^* mutant showing a femur of approximately normal length and a rudimentary zeugopod with the proximal tibia and attached putative fibula, plus a single short digit. (L) *Shh^−^*^/^*^−^*;*Dbf*/^+^ hindlimb (upside-down compared to I–K) showing a femur of normal length, full-length zeugopod and an autopod exhibiting both preaxial and postaxial polydactyly (9 triphalangeal digits, one of which is duplicated distally). Scale bars 1 mm.

As *Shh*-null embryos die perinatally, gross morphological examination of wt, *Shh^−^*^/^*^−^*, *Dbf*/^+^ and *Shh^−^*^/^*^−^*;*Dbf*/^+^ embryos was conducted at E13.5 and alcian blue staining of the limb bones was carried out at E17.5 (Fig. 2). Both forelimb and hindlimb autopods of *Shh^−^*^/^*^−^*;*Dbf*/^+^ embryos resemble those of *Dbf*/^+^ except that the broadened digital plate is more regular, shows fewer bifurcations, and is more extensive posteriorly (compare Fig. 2B, F and J with D, H and L). At E13.5 the autopod forms a 180° fan, and the angle between the autopod and zeugopod on the postaxial side of the limb is decreased to 90° (Fig. 2D, arrow).

### *2.3* *Ihh* and *Shh* expression in compound mutant limbs is mutually exclusive

**Fig. 3:**
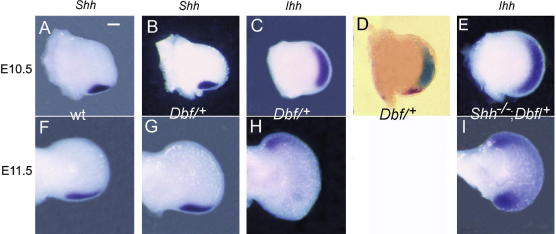
Expression of *Shh* and *Ihh* in **wt** and mutant embryo forelimb buds at E10.5 and E11.5. All darkfield except D, which shows *Shh* and *Ihh* expression on the same specimen. The *Shh* expression domain in *Dbf*/^+^ limb buds is the same as **wt** (A, B, F, G), and is mutually exclusive with that of *Ihh* (C and D). In the absence of *Shh* expression, the *Ihh* domain of compound mutant limb buds extends into the most posterior (ZPA) mesenchyme (E). Down-regulation of *Ihh* leaves a small anterior domain in *Dbf*/^+^ limb buds at E11.5 (H) but both anterior and posterior residual domains are detected in compound mutants (I). Scale bar 0.2 mm.

The expansion that characterizes the *Dbf*/^+^ autopod takes place from late E10.5 to E11.5. We therefore analysed the expression domains of *Shh* and *Ihh* in limb buds immediately prior to (E10.5) and after (E11.5) the period of expansion. In both wt and *Dbf*/^+^ limb buds at E10.5, *Shh* is expressed at the posterior margin (Fig. 3A and B), defining this region as the ZPA (Riddle et al., 1993). In wt mice *Ihh* is not expressed in limbs prior to E12.5 (St-Jacques et al., 1999) while in *Dbf*/^+^ mutant mice, *Ihh* expression is present in the distal mesenchyme of the limb bud at E10.5 (Fig. 3C). This ectopic *Ihh* domain extends throughout the area anterior to the ZPA and may correspond to the progress zone. Its absence from the ZPA was confirmed by double in situ hybridization to show nonoverlapping juxtaposed *Shh* and *Ihh* expression (Fig. 3D). Expression of *Ihh* in E10.5 *Shh^−^*^/^*^−^*;*Dbf*/^+^ mutant limb buds extends throughout the distal mesenchyme including the posterior margin, i.e. the domain in which *Shh* is expressed in *Dbf*/^+^ embryos (Fig. 3E).

At E11.5, *Shh* expression continues in the posterior margin of wt and *Dbf*/^+^ limb buds (Fig. 3F and G). Expression of *Ihh* in *Dbf*/^+^ mutant limb buds at E11.5 is progressively down-regulated from posterior to anterior, until it remains only in the anterior margin (Fig. 3H); in contrast, in *Shh^−^*^/^*^−^*;*Dbf*/^+^ mutant limbs, down-regulation of *Ihh* expression begins mid-distally, remaining strong in both the anterior and posterior mesenchyme (Fig. 3I).

### 2.4 Gene expression is altered in *Dbf* limb buds lacking *Shh*

**Fig. 4:**
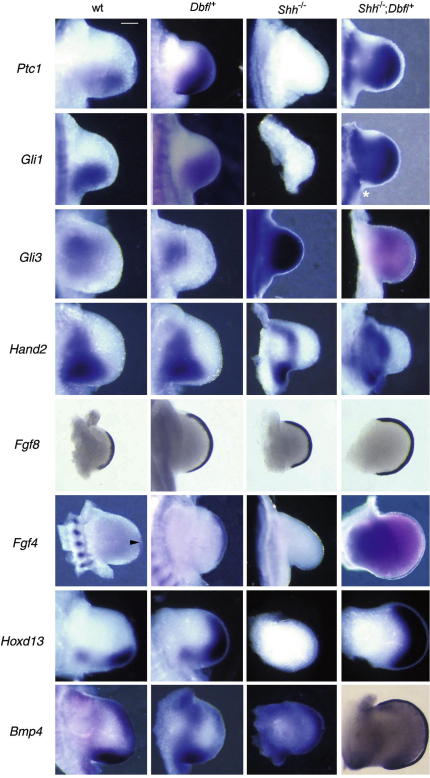
Regulation of limb development genes in E10.5 **wt**, *Dbf*/^+^;*Shh^−^*^/^*^−^* and *Shh^−^*^/^*^−^*;*Dbf*/^+^ forelimb buds. The anterior margin of limb buds is uppermost. *Ptc1* and *Gli1* expression domains are extended more anteriorly in *Dbf*/^+^ limbs than in **wt**, and are broader in *Shh^−^*^/^*^−^*;*Dbf*/^+^ limbs, but absent from *Shh^−^*^/^*^−^* limb buds. *Gli3* expression is present in the anterior region of wt limbs; the domain is reduced in *Dbf*/^+^ limbs, and extended throughout *Shh^−^*^/^*^−^* limb buds; expression is at a very low level in *Shh^−^*^/^*^−^*;*Dbf*/^+^ limb buds (extended colour development time). Expression of *Hand2* is unaltered in *Dbf*/^+^ limbs, but is present more anteriorly in *Shh^−^*^/^*^−^*;*Dbf*/^+^ limb buds. *Fgf8* is expressed throughout the AER of limb buds of each genotype. *Bmp4* expression is similar to **wt** in *Dbf*/^+^ limbs but up-regulated in *Shh^−^*^/^*^−^* limbs; in *Shh^−^*^/^*^−^*;*Dbf*/^+^ limbs expression appears to be lower in the mesenchyme but high in the AER (shown in bright field). Expression of *Hoxd13* is extended anteriorly in *Dbf*/^+^ limbs and both anteriorly and posteriorly in *Shh^−^*^/^*^−^*;*Dbf*/^+^ limbs, but is absent from *Shh^−^*^/^*^−^* limbs. *Fgf4* is expressed in the posterior region of the AER in **wt** (posterior to the arrowhead), throughout the AER in *Dbf*/^+^, is absent from *Shh^−^*^/^*^−^* and present in the anterior and posterior AER of *Shh^−^*^/^*^−^*;*Dbf*/^+^ limb buds. Scale bar 0.2 mm.

To gain insight into the mechanisms underlying the different patterns of polydactyly generated in the presence of different sources of Hh signalling in *Dbf*/^+^ and *Shh^−^*^/^*^−^*;*Dbf*/^+^ limbs, we examined the expression of genes implicated in *Shh* signalling and limb patterning in wt, *Dbf*/^+^, *Shh^−^*^/^*^−^* and *Shh^−^*^/^*^−^*;*Dbf*/^+^ limb buds at E10.5 (Fig. 4); as shown in Fig. 3, this is the stage at which *Ihh* expression is first detected. Expression of the transcriptional targets of Hh signalling, *Ptc1* and *Gli1,* is expanded anteriorly in *Dbf*/^+^ limbs; interestingly, expression of these genes is broader in the proximal mesenchyme of *Dbf/^+^*limbs lacking *Shh*, suggesting expansion of the domain of Hh signalling in these limb buds. Conversely expression of *Gli3,* which is thought to be repressed by Hh signalling (Takahashi et al., 1998), shows a reduced expression domain in *Dbf/^+^* limbs. As expected, *Gli3* is expressed throughout *Shh^−^*^/^*^−^* limbs at E10.5, but in the presence of *Ihh* in *Shh^−^*^/^*^−^*;*Dbf*/^+^ mutants it is dramatically down-regulated and required a prolonged colour development time for detection.

In wt and *Dbf*/^+^ limbs at E10.5 there is a strong expression of *Hand2* in the posterior mesenchyme, with a graded lower expression anteriorly, similar expression is seen in *Shh^−^*^/^*^−^* limbs. However, in *Shh^−^*^/^*^−^*;*Dbf*/^+^ limbs there appears to be a second strong anterior domain of *Hand2* expression, consistent with the extended expression seen at E11.5 (Fig. 1H). As reported previously (Hayes et al., 1998b; Yang et al., 1998), the *Hoxd13* domain is expanded anteriorly in *Dbf*/^+^ limb buds; in *Shh^−^*^/^*^−^*;*Dbf*/^+^ limb buds, the domain shows even greater expansion, consistent with the more regular digital fan seen in these mutants. Expression of *Fgf8* throughout the AER of expanded *Dbf*/^+^ and *Shh^−^*^/^*^−^*;*Dbf*/^+^ limbs indicates that in both mutants Hh signalling between the mesenchyme and ectodermal AER is intact. Ectopic anterior expression of *Fgf4* in the expanded limb buds of both mutants is consistent with their ectopic *Ihh* expression. *Bmp4* expression in the progress zone was slightly down-regulated in *Dbf*/^+^ limbs but up-regulated proximally; like wt limbs, it was absent from the AER. In contrast, *Shh^−^*^/^*^−^*;*Dbf*/^+^ limbs, which showed further down-regulation of *Bmp4* in the mesenchyme of the progress zone, showed ectopic expression throughout the AER. Explanation for this pattern requires further investigation.

### 2.5 The *Dbf* mutation affects the limb bud Gli3 ratio

**Fig. 5:**
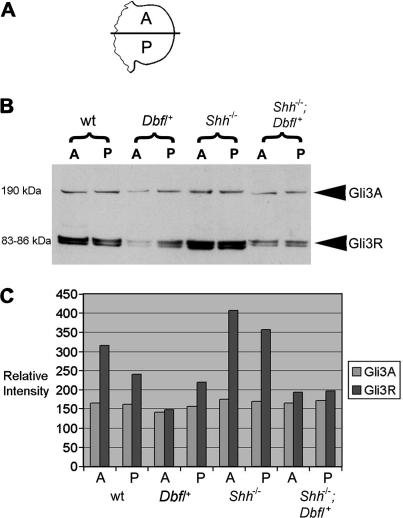
Gli3 processing is disrupted by the *Dbf*/^+^ mutation. Western blot analysis of protein extracts from the limb buds of E10.5 **wt** and mutant embryos using anti-N-terminal Gli3 antibody. (A) Representation of an E10.5 limb bud showing the anterior and posterior regions dissected for protein extraction. (B) Approximately 3 μg of protein extracted from forelimb buds was used in each lane; protein bands were immunoblotted and incubated with a Gli3-specific antibody binding to a 190 kDa band and two 83–86 kDa bands, corresponding to full-length (Gli3A) and processed repressor forms (Gli3R), respectively. A, anterior; P, posterior. (C) The histogram shows the relative intensity of each band in arbitrary units following quantification by densitometry.

The action of Gli3 protein as a transcriptional activator relies on its maintenance as Gli3A, which requires Hh signalling (Dai et al., 1999; Sasaki et al., 1999; Shin et al., 1999). To determine the effect of differential Hh signalling on Gli3 processing in mutant limb buds, we used a Gli3 antibody combined with Western blot analysis to assess the comparative levels of Gli3A and Gli3R in the anterior and posterior halves of E10.5 limb buds of all four genotypes (Fig. 5). As reported previously, wt limbs have a higher ratio of Gli3R to Gli3A anteriorly than posteriorly (Wang et al., 2000, and Fig. 5B and C). *Dbf*/^+^ limb buds have a reduced level of the repressor relative to the activator, especially in the anterior half, where levels of the two forms of Gli3 are similar. In *Shh^−^*^/^*^−^* limb buds the difference between the anterior and posterior halves is greatly reduced with relatively high levels of Gli3R to Gli3A in both halves of the limb bud mesenchyme (Litingtung et al., 2002, and Fig. 5B and C). In *Shh^−^*^/^*^−^*;*Dbf*/^+^ mutants, both halves of the limb bud show a decreased ratio of Gli3R to Gli3A compared with the wt result; the ratio is similar in both halves of the limb bud, in contrast to the *Dbf*/^+^ result which shows an A–P asymmetry.

### 2.6 A ∼600 kb deletion underlies *Dbf*

**Fig. 6:**
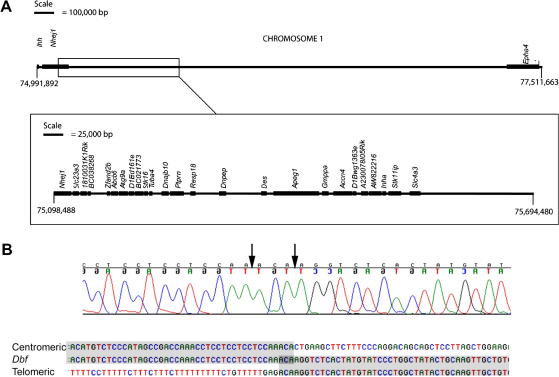
(A) Genomic map of the region around the *Dbf* deletion showing the genes flanking the deletion. The box encloses the deleted region shown at a higher scale. All nucleotide positions refer to the mm9 assembly. (B) DNA sequence chromatogram showing breakpoint sequence and alignment of this sequence compared to wt sequences on either side of the breakpoint. The arrows indicate the range of possible positions of the breakpoint within the three nucleotide identity shared by both normal sequences.

To determine the cause of the ectopic *Ihh* expression in *Dbf* limbs, we initially searched for the genetic lesion by sequencing 20 kb of the region around *Ihh* in *Dbf* heterozygotes and both parental strains, but found no pathogenic changes (data not shown). Subsequently we sought genomic rearrangements using a systematic Southern blotting strategy to interrogate the mouse genome sequence (http://genome.ucsc.edu/), which initially identified the absence of a polymorphic 8 kb SpeI fragment in *Dbf* (see Section 4.5). Characterization of the breakpoint by Southern analysis and subsequently by inverse PCR led to identification of the centromeric breakpoint at position 75,098,488 bp on chromosome 1 (Fig. 6). Analysis of sequence 3′ to this in *Dbf*/^+^ DNA revealed the telomeric breakpoint to be at position 75,694,480 bp on chromosome 1. The deleted region therefore appears to be 595,992 bp; however this figure is not precise because the deletion encompasses a ∼16 kb gap in the current mouse genome sequence (mm9 assembly) present between 75,102,130 and 75,118,131 bp. We confirmed the deletion by PCR using primers flanking the breakpoint and further demonstrated that three different loci distributed within the putatively deleted region were present only in a single copy in *Dbf*/^+^ mutant DNA (see Section 4.5). Analysis of the wt sequences at the two breakpoints showed that the sequence at the centromeric breakpoint is unique, lying within the gene *Non-homologous end joining factor 1* (*Nhej1*). However, a hexanucleotide motif CCAAAC present at the breakpoint is repeated 17 nucleotides upstream, separated by four copies of a trinucleotide CCT motif. The telomeric breakpoint resides within the 3′ terminal region of a B1 repetitive element at the endpoint of a very T-rich motif (35 thymine residues in 47 bases) which is likely to represent the complement of an ancestral poly(A) tract related to the B1 element and does not disrupt any known gene. There is a three nucleotide ambiguity in the position of the breakpoint as the sequence ACA is present on both sides of the deletion (Fig. 6). In addition to disrupting *Nhej1*, the deletion completely removes 24 known and predicted genes (Fig. 6, Supplementary Table 2 and Section 3).

### 2.7 Laurin–Sandrow syndrome does not result from large deletions 5′ of *IHH*

Laurin–Sandrow syndrome (LSS) (MIM 135750) is rare human developmental disorder characterized by triphalangeal preaxial polydactyly of the hands and feet, with variable involvement of the proximal limb elements. It has been previously suggested that LSS shares many similarities with *Dbf* and may also arise from ectopic *IHH* expression (Innis and Hedera, 2004). To investigate the possibility that *Dbf* and LSS share a common etiology, we screened five patients diagnosed with LSS for copy number variation at 23 sites between *IHH* and *EPHA4* using multiplex ligation-dependent probe amplification (see Supplementary Information). No copy number variation was detected (data not shown).

## 3 Discussion

### 3.1 Ectopic *Ihh* expression in *Dbf/^+^* is modified in the absence of *Shh* and is associated with loss of ZPA identity

Although we have previously shown that expression of ectopic *Ihh* in *Dbf*/^+^ limb buds coincides with the onset of limb bud expansion at E10.5 (Crick et al., 2003), it was not known whether the prepattern of *Dbf* limbs might be affected by the mutation prior to *Ihh* expression. However, no differences were detected in the expression of *Gli3* or *Hand2* in wt and *Dbf*/^+^ embryos at E9.5 or E10.5, consistent with the hypothesis that ectopic *Ihh* expression represents the primary pathogenic event. By E11.5, expression domains of both *Hand2* and *Gli3* were more extensive in *Dbf*/^+^ than wt limb buds, suggesting that *Ihh* signalling is able to modify their expression.

In *Dbf/^+^* limb buds, *Ihh* and *Shh* are expressed in discrete adjacent domains. Exclusion of *Ihh* from the *Shh* domain is reminiscent of the exclusion of the Hh-inducible gene *Gremlin* from this domain; Scherz et al. (2004) suggested that the effect may be due to high levels of intracellular autocrine *Shh* signalling. The loss of identity of the ZPA resulted in a abnormal expansion of the posterior limb bud mesenchyme in *Shh^−/−^*;*Dbf/^+^* mice leading to the additional postaxial polydactyly seen in these mutants.

### 3.2 Abnormal gene expression leading to an aberrant Gli3 ratio underlies *Dbf* polydactyly

To elucidate the limb patterning underlying *Dbf*/^+^ polydactyly and to investigate the generation of the broader, more regular fan of digits seen in *Shh^−^*^/^*^−^*;*Dbf*/^+^ mutants, we studied the expression of a range of limb patterning and development genes at E10.5. *Dbf*/^+^ mutant limbs show an anterior expansion of the positive regulators of Hh signalling *Ptc1*, *Gli1* and the downstream targets *Hoxd13* at E10.5 and *Hand2* by E11.5. *Dbf*/^+^ limbs also show a reduction of *Gli3* expression, which is thought to be negatively regulated by Hh signalling. Conversely, due to the complete lack of Hh activity in *Shh^−^*^/^*^−^*;*Dbf*/^+^ mutant limbs prior to E10.5, *Gli3* is ubiquitously expressed in these limb buds until this stage, when it is down-regulated in *Shh^−^*^/^*^−^*;*Dbf*/^+^ but not *Shh^−^*^/^*^−^* mutants. Gli3R is thought to repress expression of *Hoxd13* and *Hand2* and *Fgf4* in the anterior of wt limb buds while Gli3A induces the expression of *Gli1* in the posterior region (reviewed in Tickle, 2006). Therefore, the postaxial polydactyly seen in *Shh^−^*^/^*^−^*;*Dbf*/^+^ mutants may be due to the loss of identity of the ZPA with concomitant posterior extension of the *Ihh* domain. In contrast, the preaxial polydactyly that is present in both *Dbf*/^+^ and *Shh^−^*^/^*^−^*;*Dbf*/^+^ mutants is correlated with ectopic Gli3A-induced Hh signal transduction together with lack of repression of posterior patterning genes by Gli3R in the anterior of the limb bud. We suggest that the discrepancy between the very low level of *Gli3* mRNA (Fig. 4) and the Gli3 protein detected in *Shh^−^*^/^*^−^*;*Dbf*/^+^ limb buds at E10.5 (Fig. 5) indicates the perdurance of protein after the gene has been down-regulated.

### 3.3 Identification of the *Dbf* mutation

The interpretation of the mechanism of the *Dbf* mutation has been hampered previously by the failure of attempts to identify the underlying mutation. Using a Southern blotting and inverse PCR strategy we have demonstrated that a ∼600 kb deletion underlies the *Dbf* phenotype. The presence of simple sequences at both breakpoints may have predisposed them to breakage; the lack of significant similarity between the breakpoints (except for a 3 nucleotide identity at the breakpoints themselves) suggests that the rearrangement is likely to have involved nonhomologous end joining (NHEJ). Further analysis of sequence at the breakpoints revealed that the distal breakpoint resides within the degenerate poly(A)_n_ tract of a short retrotransposon (SINE) of the rodent B1 family, which, like human *Alu* repeats originate from 7Sl RNA (Vassetzky et al., 2003).

The deleted region in *Dbf* is relatively gene-dense and completely deletes 24 known and predicted transcripts as well as interrupting *Nhej1* at the centromeric breakpoint. Several of these genes have previously been implicated in abnormal mouse phenotypes or human disease; information on the known expression patterns and functions of these genes is summarized in Supplementary Table 2. Abnormalities associated with genes in the deleted region may contribute to additional aspects of the heterozygous *Dbf* phenotype such as the broadened skull, hydrocephalus, reduced viability and fertility, thickened tail and supernumerary hair follicles. However, none of the homozygous null phenotypes resulting from specific targeting of the *Ptprn*, *Des*, *Inha* or *Slc4a3* genes is lethal in late embryogenesis so the cause of death at E14.5 in *Dbf* homozygotes remains unclear. This could be attributable to loss of function of any of the genes within the interval for which homozygous mice have not yet been described, and/or to homozygosity for the ectopic *Ihh* expression defect. Interestingly a recent study reported a human fetus with a balanced *de novo* translocation t(2;7)(q36;p22) with the chromosome 2 breakpoint interrupting the orthologue of *Nhej1* at a position similar to the start of the *Dbf* deletion (Cantagrel et al., 2007). The consequence of this translocation, as in *Dbf*, would be to isolate the human *IHH* gene from possible regulatory sequences present on the opposite side of the *NHEJ1* breakpoint. Although the terminated fetus exhibited syndactyly of all four limbs, polydactyly was not present, suggesting that the translocation did not result in ectopic *IHH* expression.

We have presented evidence that the prepattern of *Dbf* limb buds is unaffected and that the preaxial polydactyly is attributable to a reduction in Gli3R resulting from ectopic *Ihh* expression. It is interesting that preaxial polydactyly, the most striking aspect of the *Dbf* phenotype, is unlikely to result directly from haploinsufficiency of any of the genes in the deleted region. Rather, the deletion appears to affect a *cis*-acting regulatory element of *Ihh*, which could be a repressor located within the deletion, or an enhancer beyond the deleted region. Other examples of regulatory mutations acting at a distance have been reviewed by Kleinjan and van Heyningen (2005). Pinpointing the regulatory sequences involved remains a major challenge, one notable success being the identification of the ZPA sequence regulatory sequence (*ZRS*) which lies ∼1.0 Mb upstream of *Shh* and regulates its expression in the ZPA; mutations in the *ZRS* lead to ectopic *Shh* expression resulting in preaxial polydactyly (Lettice et al., 2002). However, owing to the large size of the *Dbf* deletion and the large number of genes and highly conserved non-coding elements within it, it will be challenging to delineate the precise mechanism underlying ectopic *Ihh* expression in the *Dbf* mouse.

## 4 Materials and methods

### 4.1 Generation and identification of mutant mice

Mice heterozygous for the *Shh* null allele (Chiang et al., 1996) on the C57BL/6J background were mated to *Dbf*/^+^ mice on the 3H1 background. The *Shh* mutant allele was detected as previously described (Chiang et al., 1996). Homozygous *Shh*^−/−^ embryos were identified by their phenotype.

To genotype *Dbf*/^+^ embryos (prior to the identification of the causative deletion), *Dbf*/^+^ mice were crossed with wt *Mus musculus castaneus* and the *Dbf* F1 progeny were bred with C3H wt mice. Embryos were genotyped using primers which amplify the marker *D1Mit46* located ∼2.3 cM from *Ihh* (P1 5′-AGTCAGTCAGGGCTACATGATG-3′, P2 5′-CACGGGTGCTCTATTTGGAA-3′). This produces amplification products of 276 bp and 320 bp on the C3H and *Mus musculus castaneus* backgrounds respectively.

### 4.2 Whole mount *in situ* hybridization

Doubly heterozygous *Shh*^+/^*^−^*;*Dbf*/^+^ were crossed with *Shh*^+/^*^−^* mice and embryos of all six possible genotypes were collected for analysis of gene expression domains and morphology: wt, *Shh*^+/^*^−^*, *Shh^−^*^/^*^−^*, *Dbf*/^+^, *Shh*^+/^*^−^*;*Dbf*/^+^ and *Shh^−^*^/^*^−^*;*Dbf*/^+^. To ensure consistency between developmental stages, only forelimb buds were analysed and a minimum of two samples were examined with each probe. Timing of embryos was by the vaginal plug method: 12.00 noon on the day on which the plug was observed was regarded as E0.5. Pregnant females were sacrificed on the appropriate day by cervical dislocation and the embryos were dissected from the uterus in ice cold phosphate buffered saline (PBS) (140 mM NaC1, 3 mM KCl, 10 mM Na_2_HPO_4_, 2 mM KH_2_PO_4_) followed by immersion in cold tissue fixative in accordance with the Animals (Scientific Procedures) Act, 1986. Where necessary, yolk sacs were removed for genotyping and embryos were fixed by immersion overnight at 4 °C in 4% paraformaldehyde in PBS. Embryos were dehydrated by sequential washing in 25%, 50%, 75% ethanol in PBT (PBS + 0.1% Tween 20) and finally by two washes in 100% ethanol. They were stored at −20 °C until required.

Single stranded digioxygenin-UTP labelled antisense riboprobes were generated from linearized plasmids containing cDNAs. Whole mount *in situ* hybridization was carried out essentially as described by Wilkinson (1992).

### 4.3 Skeletal preparations

Embryos for skeletal staining were dissected and fixed in 95% ethanol for 1–3 days. They were immersed in alcian blue stain (75% ethanol, 20% acetic acid, 3 mg/ml alcian blue) for 21–28 days at 37 °C. They were cleared in 0.8% KOH, 20% glycerol. Following clearing, they were sequentially dehydrated and stored in 50% ethanol/50% glycerol.

### 4.4 Western Blotting

The polyclonal antibody specific for the amino terminus of Gli3 was a gift from Dr. Chin Chiang (Litingtung et al., 2002). Three μg of protein lysate derived from the anterior and posterior halves of ∼6 E10.5 forelimb buds were resolved on 4–12% polyacrylamide gels. Gli3 protein was detected using anti-N-terminal Gli3 (1:300) primary antibody and biotinylated anti-rabbit immunoglobulin-γ secondary antibody (1:1000). Protein bands were visualized by incubation with a streptavidin-peroxidase conjugate followed by an enhanced chemiluminescence detection method (Amersham).

### 4.5 Characterization of the *Dbf* deletion

To determine whether genomic rearrangements were associated with *Dbf*, single copy probes labelled with α^32^P-dCTP were synthesized and used to hybridize Southern blots of DNA isolated from heterozygous 3H1 (C3H × H101 hybrid) *Dbf* mice and wt mice from both background strains. We used the mouse genome mm9 sequence release (July 2007) for all analyses presented in this paper. A probe corresponding to 75,099,314–75,099,651 bp revealed the absence of a polymorphic 8 kb SpeI fragment in *Dbf*, found in the C3H parental strain, suggesting the existence of a deletion. A further Southern blot using a probe corresponding to 75,097,987–75,098,255 bp revealed a 1.2 kb BspHI fragment present only in *Dbf*. This 1.2 kb breakpoint fragment was isolated by inverse PCR. Briefly, genomic DNA from *Dbf* was digested with BspHI, diluted to 10 ng/μl and T4 DNA ligase was added to promote intramolecular ligation. Religated DNA was used directly in an inverse PCR using the primer pair: 5′-GCATTTGAGATTGAGACAAGCACTCTCCACAC-3′ and 5′-ACAGCGCTAGACAGAAAGCCTGCTTGCT-3′. DNA sequencing revealed 228 bp of unknown sequence that was shown by BLAST analysis to originate from a region of chromosome 1, ∼596 kb telomeric from the breakpoint. PCR amplification with primers designed either side of the breakpoint (5′-TGGTCTGGAGAGACAGCTCGTCCAGAG-3′ and 5′-GAGTTGAAGAGTTGGCATAGTGGTGCACAC-3′) was employed to confirm the site of the deletion.

To confirm the *Dbf* lesion was a true deletion, primers were designed to amplify three regions within the deletion predicted to contain polymorphisms variable between the C3H and H101 background strains. These regions were located at ∼150 kb intervals within the deleted region. The primer pairs used were site 1, 5′-GCCCTCATGCTTGAGTACCTTGCCTGTGAT-3′ and 5′-GTCCTCCCAGGGGCTGAGCAGAGTG-3′; site 2, 5′-TAGACTGAGCACCCGGCCTAACATGCTC-3′ and 5′-TGTGTCATCCACCCGGTGCCTCTGACT-3′; site 3, 5′-TAGAATTCCCACTGGGTCCACCCACTC-3′ and 5′-CATACATCCGTGTACATGTACTGACTGTCACTG-3′. Amplification products were digested with appropriate restriction endonucleases to discriminate between the alleles. Site 1 contained a novel TACC insertion polymorphism and was digested with HphI, site 2 contained a known C/T polymorphism (rs31657679) and was digested with AvaI and site 3 contained a known polymorphism (rs3049959) and was digested with *Hpy*8I. In each case only the H101 allele was present indicating that the C3H chromosome carried the deletion. The presence of both background strains was confirmed on the centromeric side of the *Dbf* deletion by sequence polymorphisms observed during Southern blotting (data not shown). Both strain backgrounds were shown to be present on the telomeric side of the deletion by AseI restriction digest of a fragment containing a novel informative C/T polymorphism which was amplified using the primer pair 5′-CAACAAAGCCCACATCAATTCACTCAGGCCGTG-3′ and 5′-CACCCTGCCTCAACCTCTCACCTGCTAG-3′.
