# Supplementary material for: Polydactyly in the mouse mutant Doublefoot involves altered Gli3 processing and is caused by a large deletion in cis to Indian hedgehog
Source: Mech Dev. 2008 May;125(5-6):517–26. doi: 10.1016/j.mod.2008.01.001 (PMC2428104; doi:10.1016/j.mod.2008.01.001)
Supplement: Supplementary data [file mmc1.doc]

**Supplementary information**

The similarities between Laurin-Sandrow syndrome (LSS) and *Dbf* have been previously highlighted and have led to the suggestion that they share a common aetiology (Innis and Hedera, 2004). Given the likelihood of *Dbf* arising from disruption of a regulatory element we screened 23 regions 5' of human *IHH* for copy number changes in 5 LSS patients using multiplex ligation dependent probe amplification (MLPA). Three of these patients were previously published (Hatchwell and Dennis, 1996; Gillessen-Kaesbach and Majewski, 1991; Matsumoto et al., 1997) the others were referred by S. Price and M. Bitner-Glindzicz.

A total of 23 conserved sites were selected to cover a total of 2.36 Mb between *IHH* and *EPHA4*. Within conserved regions, unique sites were identified by BLAST analysis and oligonucleotide probes were designed to hybridize to these sites. A full list of probes used to screen LSS patients is shown in Table 1. MLPA ligation and PCR were conducted as previously described (Schouten et al., 2002). Fragments were analysed by capillary electrophoresis using an ABI 3130 containing POP-7 polymer. Peaks were visualized using Gene Mapper v3.7 (Applied Biosystems). While we have excluded the possibility of large scale deletions 5' to *IHH* in LSS patients, we cannot rule out smaller deletions or other rearrangements in these patients.

| Probe | Sequence^a^ | Location (hg18)^a^ |
| --- | --- | --- |
| 1 | GGCCCAATATTTCCACAGCAGGATtaTAGGATCTGATTAACCATGTTAGACAACAATGCCGAGCTGG | 219644400bp-219644466bp |
| 2 | CGGAATAAAATTTATGGAGCAGCCACGAGAatTCGACCCTTTTATGTGCATGTAGAGTG | 219646799bp-219646857bp |
| 3 | GGGCAGGCGGCAGAGATCAaaGGGAGAATGCTGGAGTCAGGAG | 219654275bp-219654317bp |
| 4 | CAGGCTGACCCTACGCAACAgaACAGCACTCCAGGGTTCATCTCCAG | 219667767bp-219667813bp |
| 5 | AGGAAAGGGGAGGGGCAAGGAGGCGTGGGTTGGGAGTAtaTATGAGGTAGGCAAAGGTGAGAAAGGAGAG | 219682624bp-219682693bp |
| 6 | TTATAAATGGGACTGACCACAGCTGCCCCAcgCGCTTGGCCAGCATCATG | 219684792bp-219684841bp |
| 7 | CCCACTGCTTGCATGATTTATCCCCCTATCTGCCTGCATCGCCTttACAAGCCTCCCAGCCCTGCAGACCATTATAAAAGCTGTCTGTGCTACTG | 219690052 219690146bp |
| 8 | GCCAGAAAGCAGGTCACAATTTTTTTTATAGTCTGTTTACCAagATGCTGTTTTTACTGCCGAGGTTTCTCTCATTTTATCTCTTTTTCCC | 219702815bp-219702905bp |
| 9 | GGTGACCCCATGACCCTTCTGTGTCCTGTTTgtAGGACCCCGGGCAAGCAGAGTAACTAAAAAGATGCAGTAGTC | 219716202bp-219716276bp |
| 10 | GTCTACGCGGATTGGTGCGGGGGAAGGGGAGAAGGATGGtcCCATTCCCCCTCCATCCTGCTTTCCCTCAGCGGTCCCC | 219904994bp-219905072bp |
| 11 | GGAGGTGAGATTAGCCCCAGACTTGTCCTacTTTGGCAGAAAGTCCACAGCACTC | 219981346bp-219981400bp |
| 12 | CTTAGTAGTGTCATTTTGCATTGAGTTGACTTTGTGGGTGGTCTCCAAtaAACCCTGCACGTACAGCAAGTGTCAGCCCTGATTATC | 220321860bp-220321946bp |
| 13 | CCCGCACACTTCCCTAGCTTCTGagCTGTGTGCACCCACCAACCCGC | 219931739bp-219931785bp |
| 14 | CTTTTATTCAGGAAGCAAGGAGGCtaCATTGGGGAATGGTGACTTTGGTTC | 219776748bp-219776797bp |
| 15 | GGGAAATGCTTTCATCCCTGATGAttCTGAGCCAAAGTACAATTGCCTCCTTCAG | 219847564bp-219847618bp |
| 16 | CGGGAGGCTAAATGAGGGAGAttATCACCCAACTGCAGCCGGG | 220084794bp-220084836bp |
| 17 | GAGTTGGCAGTTACTGTTTGGGAGAatCTGCCCTTCTAAGTTCCAGATGATTAATAAAC | 220168686bp-220168744bp |
| 18 | CTTTCCTGGTAATTATTCAGTAATTATTCACATAAGAAGGATTTCAttGTTATATTTCATATATTCGTTGTAGTTGATGGCCTTTTAATAGTGAC | 220344998bp-220345092bp |
| 19 | CAAAAGAGGATTGTGAATATTAAAAAGCAGCCCAGCCAGTTAGAGTTaaACCGTCGGGATAAGATATTAACTTAAACCACATGTGAAGTGC | 220480013bp-220480103bp |
| 20 | GGAAAAACAACAACAAAAGTCTACCAAAGGACAGGATtaAGTGAAATTTCTTAAACAGATTTTTAAAACCGTTGTGAAC | 220734874bp-220734952bp |
| 21 | CATTAAAAAAGAAAGAGAAATTGATTTGAAGACGCAAGATGGGATTGAGttGTAATATAAAAGTTGCTGGTCTTTTACTCCTGAGAG | 221064544-bp221064630bp |
| 22 | GTAAAACTACATCTGGAAATGACAAGCACACAGTTGTAaaTCTGAAAGTCAGTGTGTTGTTGTAATAGCTTGCAG | 221454326bp-221454400bp |
| 23 | GATTAGCACAAACATGTTGGATTTTGCGAACATACCTtaAGACTTTATTAGCCTACCTTATTTTATATGTC | 221670128bp-221670198bp |

**Table 1 Sequences of the 23 regions assayed for copy number variations by MLPA in Laurin Sandrow syndrome.**

a. Ligation sites are in lower case and the location of each probe in the human genome is also given (all nucleotide positions refer to the hg18 build)

| **Gene** | **Description** | **Function** | **Alleles**^1^ |
| --- | --- | --- | --- |
| *NheJ1* | Nonhomologous end-joining factor 1 | DNA repair | Truncation of *NHEJ1* associated with polymicrogyria (Cantagrel et al., 2007). Mutations in *NHEJ1* associated with growth retardation, microcephaly, and immunodeficiency (Buck et al., 2006). Murine ES cells lacking *Nhej1* are highly sensitive to ionizing radiation and show increased genomic instability (Zha et al., 2007). |
| *Slc23a3* | Solute carrier 23 | Membrane located permease |  |
| *1810031K17Rik* | Hypothetical protein LOC69171 | Unknown |  |
| *BC038286* | Hypothetical Protein C2orf17 homolog | Multipass membrane protein (predicted) |  |
| *Zfand2b* | Zinc finger, AN1 type domain 2B | DNA binding |  |
| *Abcb6* | ATP-binding casette, sub-family B | Heme biosynthesis | Member of ATP-binding cassette (ABC) superfamily, many members of which are involved in human disease (Allikmets et al., 1997). *Abcb6* is a highly expressed in liver and is a heme/protoporphyrin importer reviewed in (Krishnamurthy et al., 2006). |
| *Atg9a* | Autophagy-related 9-like 1 | Autophagy (by similarity) |  |
| *Stk16* | Serine Threonine kinase 16 | Protein kinase activity | Ubqiuitously expressed at low levels, localized in Golgi. |
| *Tuba4* | Tubulin, alpha 4 | Component of microtubules |  |
| *Dnajb10* | DNAJ homolog, subfamily B, member 10 | Heat shock protein binding |  |
| *Ptprn* | Receptor-type tyrosine-protein Neuroendocrine secretion phosphatase-like N precursor | Neuroendocrine secretion | Homozygous mice show decreased insulin response to glucose (Saeki et al., 2002). |
| *Resp18* | Regulated endocrine-specific protein 18 | May regulate corticotrophs | Present in blood pressure quantitative trait locus in rat genome (Garrett et al., 2005) |
| *Dnpep* | Aspartyl aminopeptidase | Intracelllular peptide metabolism |  |
| *Des* | Desmin | Muscle cell intermediate filament | Homozygous null mice show smooth muscle and heart defects (Li et al., 1996). *DES* mutations found in patients with cardioskeletal myopathy, myofibrillar myopathy and cardiomyopathy (Dalakas et al., 2000; Goldfarb et al., 1998). |
| *Apeg1* | Aortic preferentially expressed protein 1 | Arterial smooth muscle cell development | Genomic rearrangements at *APEG1* locus associated with atherosclerosis (Arvanitis et al., 2005*). APEG1* is downregulated by vascular injury (Hsieh et al., 1996) |
| *Gmppa* | GDP-mannose pyrophosphorylase A | Nucleotidyltransferase activity |  |
| *Accn4* | Amiloride-sensitive cation channel 4 | Membrane transport |  |
| *D1Bwg1363e* | Chondroitin polymerizing factor isoform a | Biosynthesis |  |
| *A230078I05Rik* | Hypothetical protein LOC319998 | Unknown |  |
| *AW822216* | Hypothetical protein LOC98733 | Unknown |  |
| *Inha* | Inhibin alpha chain precursor | TGF-beta family signaling | *Inha* deficient mice develop tumors in testis and adrenal gland (Matzuk et al 1992;1994). Loss of heterozygosity also found in 42% of human prostate cancers (Schmitt et al., 2002). |
| *Stk11ip* | Serine/threonine kinase 11 interacting protein | Signalling | Interacts with Stk11, mutations of which underlie the majority of Peutz—Jeghers syndrome cases (reviewed in Udd et al., 2004) |
| *Slc4a3* | Anion exchange protein 3 | Plasma membrane anion exchange | Mice homozygous for a targeted disruption of *Slc4a3* show a reduced seizure threshold (Hentschke et al., 2006) |

**Table 2. Summary of known information regarding genes regarding genes deleted or disrupted by the *Dbf* deletion.**

^1^ Information on murine alleles was obtained from Mouse Genome Informatics database (<http://www.informatics.jax.org/>) and on human alleles from Online Mendelian Inheritance in Man (www.ncbi.nlm.nih.gov/omim).

**
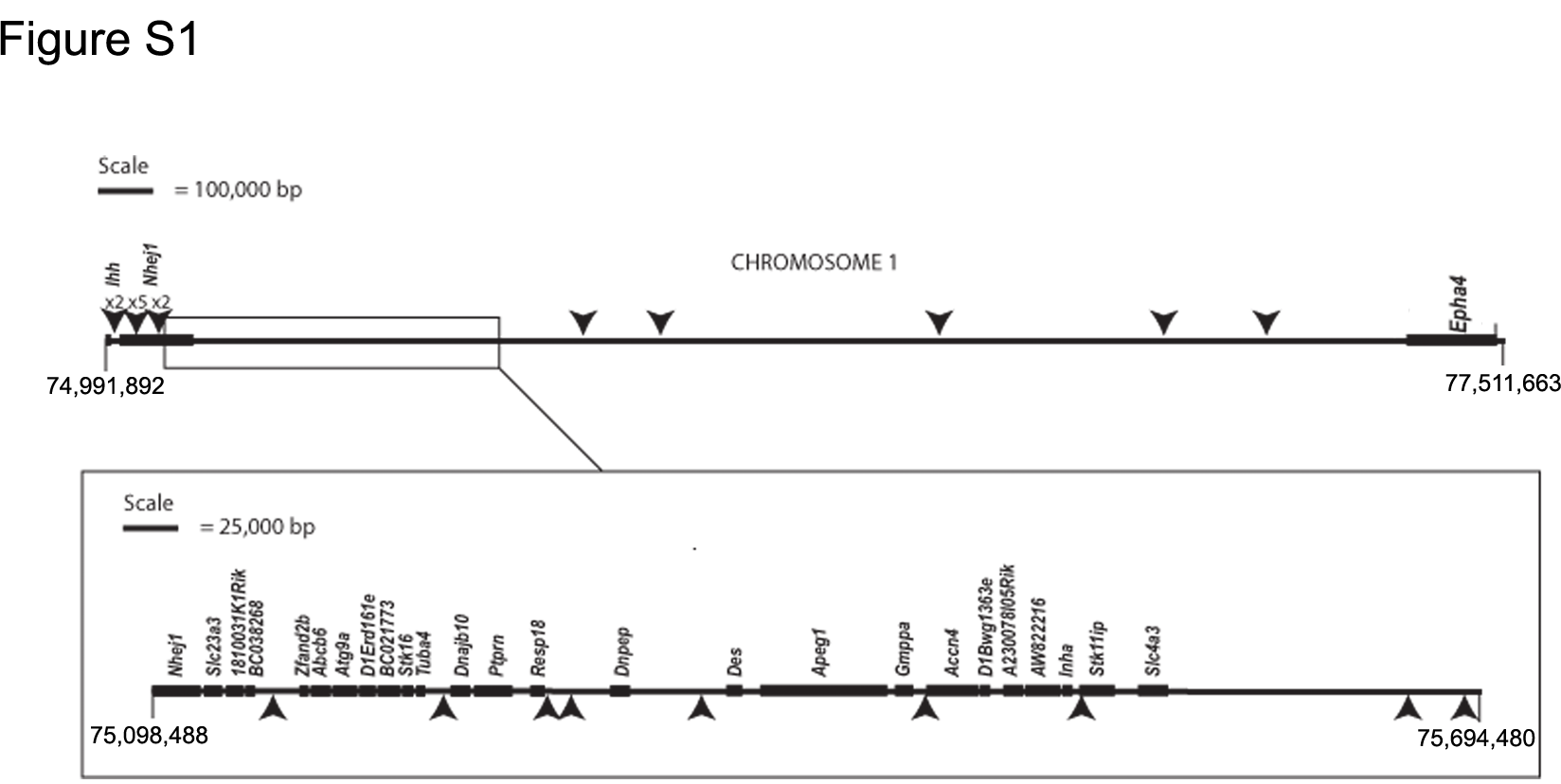
**

**Figure S1.** Genomic map of the region around the *Dbf* deletion on mouse chromosome 1 showing the genes flanking the deletion. The box encloses the deleted region shown at a higher scale. All nucleotide positions refer to the mm9 assembly. The arrowheads indicate the equivalent regions of the mouse genome screened in Laurin Sandrow patients (for locations of these probes in the human genome see Table 1).

**Supplementary Information References**

Allikmets, R., Shroyer, N.F., Singh, N., Seddon, J.M., Lewis, R.A., Bernstein, P.S., Peiffer, A., Zabriskie, N.A., Li, Y., Hutchinson, A., Dean, M., Lupski, J.R. and Leppert, M. (1997) Mutation of the Stargardt disease gene (*ABCR*) in age-related macular degeneration. Science 277, 1805-1807.

Arvanitis, D.A., Flouris, G.A. and Spandidos, D.A. (2005) Genomic rearrangements on *VCAM1*, *SELE*, *APEG1*and *AIF1* loci in atherosclerosis. Journal of Cellular & Molecular Medicine 9, 153-159.

Buck, D., Malivert, L., de Chasseval, R., Barraud, A., Fondaneche, M.C., Sanal, O., Plebani, A., Stephan, J.L., Hufnagel, M., le Deist, F., Fischer, A., Durandy, A., de Villartay, J.P. and Revy, P. (2006) *Cernunnos*, a novel nonhomologous end-joining factor, is mutated in human immunodeficiency with microcephaly. Cell 124, 287-299.

Cantagrel, V., Lossi, A.M., Lisgo, S., Missirian, C., Borges, A., Philip, N., Fernandez, C., Cardoso, C., Figarella-Branger, D., Moncla, A., Lindsay, S., Dobyns, W.B. and Villard, L. (2007) Truncation of *NHEJ1* in a patient with polymicrogyria. Human Mutation 28, 356-364.

Dalakas, M.C., Park, K.Y., Semino-Mora, C., Lee, H.S., Sivakumar, K. and Goldfarb, L.G. (2000) Desmin myopathy, a skeletal myopathy with cardiomyopathy caused by mutations in the *DESMIN* gene. N Engl J Med 342, 770-780.

Garrett, M.R., Meng, H., Rapp, J.P. and Joe, B. (2005) Locating a blood pressure quantitative trait locus within 117 kb on the rat genome: substitution mapping and renal expression analysis. Hypertension 45, 451-459.

Gillessen-Kaesbach, G. and Majewski, F. (1991) Bilateral complete polysyndactyly (type IV Haas). American Journal of Medical Genetics 38, 29-31.

Goldfarb, L.G., Park, K.Y., Cervenakova, L., Gorokhova, S., Lee, H.S., Vasconcelos, O., Nagle, J.W., Semino-Mora, C., Sivakumar, K. and Dalakas, M.C. (1998) Missense mutations in *DESMIN* associated with familial cardiac and skeletal myopathy. Nat Genet 19, 402-403.

Hatchwell, E. and Dennis, N. (1996) Mirror hands and feet: a further case of Laurin-Sandrow syndrome. Journal of Medical Genetics 33, 426-428.

Hentschke, M., Wiemann, M., Hentschke, S., Kurth, I., Hermans-Borgmeyer, I., Seidenbecher, T., Jentsch, T.J., Gal, A. and Hubner, C.A. (2006) Mice with a targeted disruption of the Cl-/HCO3- exchanger *Ae3* display a reduced seizure threshold. Molecular & Cellular Biology 26, 182-191.

Hsieh, C.M., Yoshizumi, M., Endege, W.O., Kho, C.J., Jain, M.K., Kashiki, S., de los Santos, R., Lee, W.S., Perrella, M.A. and Lee, M.E. (1996) *APEG-1*, a novel gene preferentially expressed in aortic smooth muscle cells, is down-regulated by vascular injury. Journal of Biological Chemistry 271, 17354-17359.

Innis, J.W. and Hedera, P. (2004) Two patients with monomelic ulnar duplication with mirror hand polydactyly: segmental Laurin-Sandrow syndrome. Am J Med Genet A 131, 77-81.

Krishnamurthy, P.C., Du, G., Fukuda, Y., Sun, D., Sampath, J., Mercer, K.E., Wang, J., Sosa-Pineda, B., Murti, K.G. and Schuetz, J.D. (2006) Identification of a mammalian mitochondrial porphyrin transporter. Nature 443, 586-589.

Li, Z., Colucci-Guyon, E., Pincon-Raymond, M., Mericskay, M., Pournin, S., Paulin, D. and Babinet, C. (1996) Cardiovascular lesions and skeletal myopathy in mice lacking *Desmin*. Dev Biol 175, 362-366.

Matsumoto, N., Ohashi, H., Kato, R., Fujimoto, M., Tsujita, T., Sasaki, T., Nakano, M., Miyoshi, O., Fukushima, Y. and Niikawa, N. (1997) Molecular mapping of a translocation breakpoint at 14q13 in a patient with mirror-image polydactyly of hands and feet. Human Genetics 99, 450-453.

Matzuk, M.M., Finegold, M.J., Mather, J.P., Krummen, L., Lu, H. and Bradley, A. (1994) Development of cancer cachexia-like syndrome and adrenal tumors in inhibin-deficient mice. Proc Natl Acad Sci U S A 91, 8817-8821.

Matzuk, M.M., Finegold, M.J., Su, J.G., Hsueh, A.J. and Bradley, A. (1992) *Alpha-inhibin* is a tumour-suppressor gene with gonadal specificity in mice. Nature 360, 313-319.

Saeki, K., Zhu, M., Kubosaki, A., Xie, J., Lan, M.S. and Notkins, A.L. (2002) Targeted disruption of the protein tyrosine phosphatase-like molecule IA-2 results in alterations in glucose tolerance tests and insulin secretion. Diabetes 51, 1842-1850.

Schmitt, J.F., Millar, D.S., Pedersen, J.S., Clark, S.L., Venter, D.J., Frydenberg, M., Molloy, P.L. and Risbridger, G.P. (2002) Hypermethylation of the inhibin alpha-subunit gene in prostate carcinoma. Molecular Endocrinol 16, 213-220.

Schouten, J.P., McElgunn, C.J., Waaijer, R., Zwijnenburg, D., Diepvens, F. and Pals, G. (2002) Relative quantification of 40 nucleic acid sequences by multiplex ligation-dependent probe amplification. Nucleic Acids Res 30, e57.

Udd, L., Katajisto, P., Rossi, D.J., Lepisto, A., Lahesmaa, A.M., Ylikorkala, A., Jarvinen, H.J., Ristimaki, A.P. and Makela, T.P. (2004) Suppression of Peutz-Jeghers polyposis by inhibition of cyclooxygenase-2. Gastroenterology 127, 1030-1037.

Zha S, A.F.W., Cheng H.L., Brush J.W. and Li G (2007) Defective DNA repair and increased genomic instability in Cernunnos-XLF-deficient murine ES cells. Proc Natl Acad Sci U S A 104, 4518-4523.
